# Diagnostic delay of veno-occlusive disease in a myelofibrosis patient with pre-transplant transjugular intrahepatic portosystemic shunt: a case report

**DOI:** 10.3389/fonc.2026.1820815

**Published:** 2026-07-28

**Authors:** Cristina Rotolo, Roberto Bono, Giuseppe Sapienza, Corinne Spoto, Roberto Miraglia, Luca Castagna

**Affiliations:** 1 Unità Bone Marrow Transplantation Unit (BMT), Azienda Ospedaliera Villa Sofia-Cervello, Palermo, Italy; 2 Radiology Service, Department of Diagnostic and Therapeutic Services, Mediterranean Institute for Transplantation and Advanced Specialized Therapies (ISMETT), Palermo, Italy

**Keywords:** allogeneic stem cell transplantation, myelofibrosis, sinusoidal obstruction syndrome, tips, veno-occlusive disease

## Abstract

Veno-occlusive disease (VOD), also known as sinusoidal obstruction syndrome (SOS), is a life-threatening complication of allogeneic hematopoietic stem cell transplantation (allo-HSCT) characterized by endothelial injury and post-sinusoidal portal hypertension. Classical diagnostic criteria rely on clinical signs such as ascites, weight gain, and painful hepatomegaly; however, these features may be altered in patients with modified portal hemodynamics. We report the case of a 46-year-old woman with primary myelofibrosis who underwent transplantation after placement of a transjugular intrahepatic portosystemic shunt to manage severe portal hypertension. To our knowledge, this is the first reported case of VOD occurring after transplantation in a patient with a pre-existing transjugular intrahepatic portosystemic shunt, representing a unique diagnostic challenge. Early after transplantation, the patient developed rapidly progressive hyperbilirubinemia without the typical manifestations of VOD, leading initially to an alternative diagnostic hypothesis and delayed initiation of defibrotide therapy. The presence of the shunt likely attenuated hallmark clinical signs by decompressing the portal venous system, thereby masking the syndrome until advanced liver failure occurred. Despite treatment, the patient experienced progressive hepatic dysfunction and died. This case highlights a previously underrecognized diagnostic pitfall: standard diagnostic criteria for VOD may be unreliable in patients with altered portal circulation. Clinicians should maintain a high index of suspicion in this setting and consider early treatment when biochemical abnormalities emerge, as prompt recognition remains critical for improving outcomes.

## 1 Introduction

Primary myelofibrosis (PM) is a chronic myeloproliferative neoplasm characterized by clonal proliferation of hematopoietic stem cells, progressive bone marrow fibrosis, extramedullary hematopoiesis, and splenomegaly (1). In advanced phases, patients may develop non-cirrhotic portal hypertension, frequently associated with thrombosis of the splanchnic venous system and complicated by esophageal varices or refractory ascites. In selected cases, decompression with a transjugular intrahepatic portosystemic shunt (TIPS) is required (2).

Although the introduction of JAK/STAT pathway inhibitors, such as ruxolitinib, has significantly improved symptom control and quality of life, allogeneic hematopoietic stem cell transplantation (allo-HSCT) remains the only potentially curative treatment options (3).

Allo-HSCT is typically indicated for patients classified as intermediate-2 or high risk according to the Dynamic International Prognostic Scoring System (DIPSS) (4) or (DIPSS-plus) (5), as well as for those with high-risk genetic mutations (such as *ASXL1*, *EZH2*, *SRSF2*, *IDH1/2*) (6, 7).

Among the most severe hepatic complications of allo-HSCT is veno-occlusive disease (VOD), also known as sinusoidal obstruction syndrome (SOS) (8). It results from endothelial injury of hepatic sinusoids, causing sinusoidal narrowing, post-sinusoidal portal hypertension, and hepatocellular necrosis. Typical manifestations include painful hepatomegaly, rapid weight gain, ascites, and hyperbilirubinemia, with high mortality rates in severe cases (9). Medical therapy with defibrotide is successful in most patients (10). At our institution, VOD is diagnosed and graded according to the updated European Society for Blood and Marrow Transplantation (EBMT) criteria. Defibrotide is recommended in patients with a confirmed diagnosis of severe VOD with an up dose of drug in case of very severe VOD.

However, these diagnostic criteria may be unreliable in patients with altered portal hemodynamics. To our knowledge, no cases of VOD occurring after transplantation in patients with a pre-existing transjugular intrahepatic portosystemic shunt (TIPS) have been previously reported. We describe a case in which the presence of TIPS masked classical clinical signs, contributing to delayed diagnosis and poor outcome.

## 2 Case description

A 46-year-old woman with PM with the JAK2V617F mutation, intermediate-2 risk by DIPSS, was referred for allo-HSCT from an HLA-identical sibling donor. The disease was diagnosed in October 2020 following symptomatic splenomegaly (16.5 cm) and thrombosis involving the portal vein, left subclavian vein, and right internal jugular vein. Ruxolitinib was administered from January 2021 to December 2021 but was discontinued due to intolerance and lack of efficacy. Subsequently, she was enrolled in a clinical trial with Navitoclax plus Ruxolitinib until May 2024. At that time, ultrasound documented progressive splenomegaly (19 cm), indicating disease progression despite therapy. Consequently, Fedratinib was initiated in June 2024. In November 2024, refractory ascites and portal biliopathy secondary to chronic splanchnic venous thrombosis were diagnosed. To decongest the portal system, a TIPS was placed, lowering the portosystemic gradient from 22 mmHg to 7 mmHg. The procedure was complicated by an episode of portosystemic encephalopathy requiring hospitalization. Pre-transplant hepatic function was preserved despite partial diversion of portal flow through the shunt. Notably, the pre-existing TIPS was essential to make transplantation feasible, as it significantly reduced hypersplenism-related complications such as refractory ascites and portal hypertension, which would otherwise have precluded allo-HSCT. The patient underwent allo-HSCT with reduced-intensity conditioning (RIC) using treosulfan 30 g/m2 and fludarabine 160 mg/m2 (FT10 regimen). GvHD prophylaxis consisted of cyclosporine, short-course methotrexate, and anti-thymocyte globulin (ATG 5 mg/kg) day -2 and -1. Liver stiffness was monitored by transient elastography (FibroScan^®^), showing a baseline value of 5.0 kPa before conditioning and 7.6 kPa on day +5, when a rapid increase in total bilirubin (3.3 mg/dL) was observed without other signs suggestive of VOD/SOS. Serial weekly assessments were subsequently performed over the following two weeks as part of routine clinical monitoring. Transaminases were mildly elevated, and the patient experienced mild encephalopathy. An extensive diagnostic work-up was performed to investigate alternative causes of liver dysfunction. Viral reactivation, including cytomegalovirus (CMV), Epstein–Barr virus (EBV), human herpesvirus 6 (HHV-6), and hepatitis viruses, was excluded by virological testing. Transplant-associated thrombotic microangiopathy (TA-TMA) was also investigated and considered unlikely based on the overall clinical and laboratory findings. Doppler ultrasonography confirmed TIPS patency, making shunt dysfunction an unlikely explanation for the progressive liver dysfunction. Given the absence of the typical clinical features of VOD/SOS and the stable liver stiffness findings, drug-induced liver injury was initially considered the most likely diagnosis. At day +8, due to progressive hyperbilirubinemia (8.5 mg/dL, peaking at 21.4 mg/dL on day +21) and clinical deterioration, defibrotide was initiated at 25 mg/kg/day. Hepatic venous pressure gradient (HVPG)-the gold standard for assessing sinusoidal congestion and portal hypertension-was not performed because the presence of the TIPS, which artificially decompresses the portal venous system, would have inevitably rendered the value non-interpretable. Despite therapy, hepatic failure progressed, and the patient died.

## 3 Discussion

VOD/SOS is one of the most severe complications of allo-HSCT, with an incidence of 5%-15% and a mortality rates historically exceeding 80% in untreated severe cases complicated by multiorgan dysfunction (9, 11). Risk factors for VOD include patient-related factors (pre-existing liver disease; iron overload with elevated ferritin), disease-related factors (myeloproliferative neoplasm), and treatment-related exposures (hepatotoxic agents; intensity of conditioning regimen). The coexistence of multiple risk factors markedly increases susceptibility and worsens prognosis.

Diagnostic criteria have evolved over time: from the Seattle to the Baltimore criteria, and, lastly, to the EBMT consensus (11, 12). The 2016 EBMT criteria (updated 2023) introduced several refinements: distinction between early-onset (≤21 days) and late-onset (>21 days) VOD, integration of Doppler ultrasound and elastography, and a severity grading system incorporating organ dysfunction (13). None of these criteria were developed or validated in populations with altered portal hemodynamics due to TIPS.

According to the updated EBMT diagnostic criteria, a diagnosis of VOD/SOS in adult patients requires a serum bilirubin level ≥2 mg/dL in combination with at least two additional features, including ascites, painful hepatomegaly, weight gain exceeding 5% of baseline body weight, and/or hemodynamic or imaging findings consistent with sinusoidal obstruction syndrome. In the present case, the patient initially exhibited isolated hyperbilirubinemia without any of the accompanying clinical, hemodynamic, or ultrasonographic features required to establish the diagnosis. Consequently, VOD/SOS was not considered sufficiently supported at that stage according to current diagnostic criteria. Furthermore, alternative etiologies of liver dysfunction, particularly drug-induced liver injury related to the conditioning regimen and concomitant medications, were initially regarded as more likely explanations for the observed bilirubin elevation. Only with the subsequent progressive increase in bilirubin levels and clinical deterioration did the suspicion of VOD/SOS become stronger, ultimately leading to the initiation of defibrotide therapy.

To our knowledge, no cases of patients undergoing allo-HSCT with a pre-existing TIPS have been reported in the literature. All published reports describe TIPS placement as a therapeutic intervention for established VOD/SOS, rather than a pre-transplant condition. This makes the present case unique and further complicates the diagnostic interpretation, as none of the existing diagnostic criteria have been validated in this setting.

In the present case, the presence of a TIPS profoundly altered the hemodynamic consequences of early VOD/SOS. By decompressing the portal venous system and reducing sinusoidal pressures, TIPS may attenuate or abolish hallmark signs such as ascites, weight gain, and painful hepatomegaly. Consequently, bilirubin elevation may be the only detectable feature, delaying diagnosis until advanced liver failure ensues. This mechanism likely contributed to the late recognition of VOD in our case, leading to a delay in defibrotide initiation at a stage when therapeutic benefit is limited; indeed, defibrotide’s benefit is time dependent. Delayed initiation (3 days after the onset of bilirubin elevation) is associated with significantly poorer survival (14), which likely contributed to the fatal outcome of this patient.

## 4 Conclusion

This case highlights that classical diagnostic criteria may not be applicable in patients with altered portal hemodynamics, and that modified diagnostic strategies explicitly validated in patients with TIPS are required. In patients undergoing allo-HSCT with a pre-existing TIPS, VOD may present without classical signs due to altered portal hemodynamics. Clinicians should maintain a high index of suspicion for VOD, even in the absence of ascites, weight gain, or hepatomegaly. Early recognition and prompt initiation of defibrotide are essential, as diagnostic delay significantly worsens outcomes.

**Figure 1:**
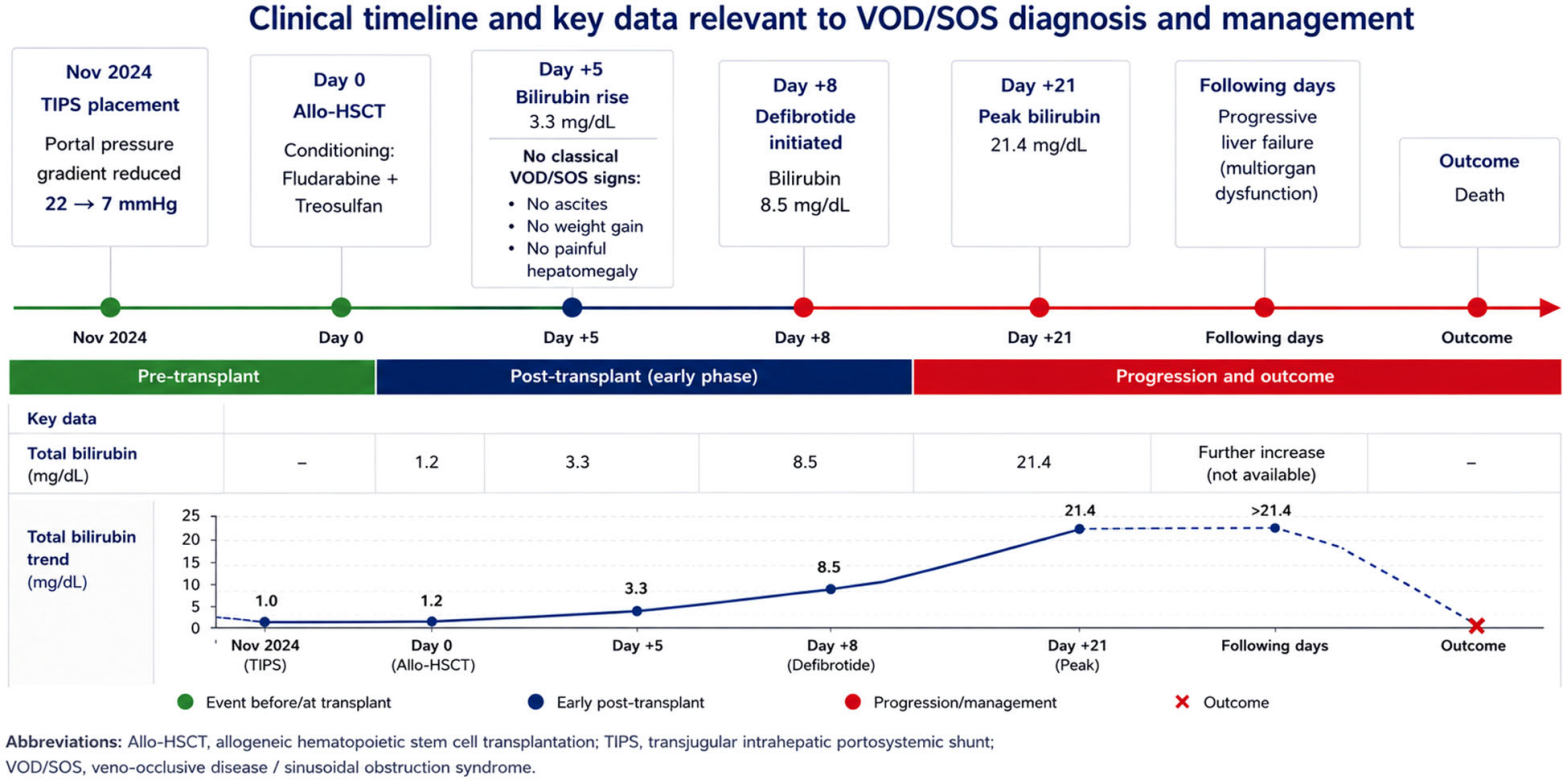
Clinical timeline showing the patient's course, key events related to VOD/SOS diagnosis and management, bilirubin trends, treatment with defibrotide, and clinical outcome.

## Data Availability

The raw data supporting the conclusions of this article will be made available by the authors, without undue reservation.
